# A Comparative Approach to Understanding Tissue-Specific Expression of Uncoupling Protein 1 Expression in Adipose Tissue

**DOI:** 10.3389/fgene.2012.00304

**Published:** 2013-01-03

**Authors:** Andrew Shore, Richard D. Emes, Frank Wessely, Paul Kemp, Clemente Cillo, Maria D’Armiento, Nigel Hoggard, Michael A. Lomax

**Affiliations:** ^1^School of Biosciences, Cardiff UniversityCardiff, UK; ^2^School of Veterinary Medicine and Science, University of Nottingham, Sutton Bonington CampusLeicestershire, UK; ^3^Faculty of Medicine, National Heart and Lung Institute, Imperial College LondonLondon, UK; ^4^Department of Clinical and Experimental Medicine, Federico II University Medical SchoolNaples, Italy; ^5^Department of Biomorphological and Functional Sciences, Federico II University Medical SchoolNaples, Italy; ^6^Rowett Institute of Nutrition and Health, Aberdeen Centre for Energy Regulation and Obesity, University of AberdeenAberdeen, UK; ^7^School of Biosciences, University of Nottingham, Sutton Bonington CampusLeicestershire, UK

**Keywords:** CpG islands, methylation, uncoupling protein 1, phylogenic analysis

## Abstract

The thermoregulatory function of brown adipose tissue (BAT) is due to the tissue-specific expression of uncoupling protein 1 (UCP1) which is thought to have evolved in early mammals. We report that a CpG island close to the UCP1 transcription start site is highly conserved in all 29 vertebrates examined apart from the mouse and xenopus. Using methylation sensitive restriction digest and bisulfite mapping we show that the CpG island in both the bovine and human is largely un-methylated and is not related to differences in UCP1 expression between white and BAT. Tissue-specific expression of UCP1 has been proposed to be regulated by a conserved 5′ distal enhancer which has been reported to be absent in marsupials. We demonstrate that the enhancer, is also absent in five eutherians as well as marsupials, monotremes, amphibians, and fish, is present in pigs despite UCP1 having become a pseudogene, and that absence of the enhancer element does not relate to BAT-specific UCP1 expression. We identify an additional putative 5′ regulatory unit which is conserved in 14 eutherian species but absent in other eutherians and vertebrates, but again unrelated to UCP1 expression. We conclude that despite clear evidence of conservation of regulatory elements in the UCP1 5′ untranslated region, this does not appear to be related to species or tissues-specific expression of UCP1.

## Introduction

In eutherians, non-shivering thermogenesis (NST) occurs in brown adipose tissue (BAT) which expresses a tissue-specific gene, uncoupling protein 1 (UCP1; Cannon and Nedergaard, 2004). This gene codes for a mitochondrial protein with the ability to uncouple oxidative phosphorylation and generate heat. Recently BAT has been identified in adult humans and has been suggested to offer a potential target to increase energy expenditure and treat obesity(Nedergaard et al., 2007).

The expression of UCP1 is cell-specific to brown adipocytes and has been identified in all mammalian neonates so far examined except the pig, in which exons 3–5 were deleted about 20 million years ago (Berg et al., 2006). BAT-specific UCP1 expression is a feature of small mammals, hibernators, and newborns and is thought to have originated prior to the Eutherian mammal radiation as it has been found in the rock elephant shrew, a member of the Afrotherian mammalian lineage (Mzilikazi et al., 2007). Recent discoveries of UCP1 in non-eutherian marsupials, and of UCP1 orthologs in the non-mammalian vertebrates, frogs, and fish, expressed in liver and muscle, respectively, have questioned this view (Klingenspor et al., 2008; Hughes et al., 2009). Phylogenetic analysis has demonstrated rapid evolution of UCP1 on the Eutherian lineage and suggested that a model of relaxed constraints as predicted from the coevolution of genes which have taken over some of UCP1 function, rather than directional selection, seems to be involved (Hughes et al., 2009). Evidence to support a role of the UCP2 and 3 in oxidative stress suggests that subfunctionalization of these paralogs allowed the divergence of the BAT-specific expression of UCP1 and its role in NST (Klingenspor et al., 2008).

Most newborn mammals are particularly vulnerable to hypothermia, and NST in BAT plays an important role depending on the thermoregulatory behavior of different mammals (Symonds and Lomax, 1992). In altricious newborn such as rodents, pups are born blind and naked, and require the protection of a nest environment to prevent hypothermia until BAT becomes active a few days after birth (Cannon and Nedergaard, 2004). Immature newborns (e.g., hamster) only recruit NST in BAT a week or more after birth with marsupials being an extreme group of immature mammals who do not develop independent NST until the young need to leave the pouch. In contrast to altricious and immature newborns, in precocious mammals (e.g., cows and sheep), BAT develops during fetal life with maximal thermogenic activity occurring immediately after birth to allow the newborn to quickly achieve independent thermoregulation (Symonds and Lomax, 1992). Human fetuses and neonates also possess BAT and fit best into the precocial group (Cannon and Nedergaard, 2004) although BAT has been identified in adult humans (McKinnon and Docherty, 2001).

The exact mechanism which confers BAT-specific expression of UCP1 is not known. Studies on the rodent promoter have revealed a highly conserved 221 bp enhancer element located approximately –2.5 kb from the transcriptional start that confers both hormonal and tissue-specific responses (Cassard-Doulcier et al., 1998). The enhancer unit is also highly conserved across a 5 kb genomic sequence upstream of the UCP1 transcription start site in eutherians, including the Afrotherian species but could not be found in marsupials, despite cold-induced UCP1 expression in BAT (Hughes et al., 2009). In a recent study we have proposed that tissue-specific expression may be dictated by the methylation of CpGs in cyclic AMP response elements in the enhancer unit (Shore et al., 2010). Methylation of CpGs in CpG islands (CGI) in the promoter may also confer tissue-specific expression of UCP1 (Kiskinis et al., 2007). Alternatively, tissue-specific expression of UCP1 during development may be governed by the expression of transcriptional regulators as reported in our previous studies (Lomax et al., 2007).

CpGs are generally methylated in the genome except where they occur in CGI around the start of transcription of genes (Sakurai et al., 2006). These CGI, are a feature of TATA-less promoters, and can act as strong promoters of transcription, this effect being modulated by the degree of CpG methylation. Identification of regions of genomic DNA that have been conserved across divergent species is a commonly used method of indicating important regulatory elements.

Here we employ bioinformatic and molecular approaches to demonstrate that despite evidence of conservation of a CpG island, as well as regulatory elements, in the UCP1 promoter in mammals and vertebrates, these are insufficient to explain expression differences between mammalian species and tissues.

## Materials and Methods

### Tissues

Bovine perirenal brown fat was obtained from a 1-day-old male calf. Human fetal samples were obtained from legally approved therapeutic terminations at the Department of Pathology University of Naples Federico II under the control of the University’s Guidelines for Human Experimentation. Informed consent was obtained from all the subjects involved in the experiments and the study protocols were reviewed and approved by the University Ethical Committee. The age of the fetuses was calculated from anamnesis and ultrasonographic data, to be in the range from 22 to 34 gestational weeks. Tissues were dissected, typically within 2 h after death. The biopsies of perirenal fetal BAT were immediately frozen in liquid nitrogen and then stored in a freezer at −80°C until DNA/RNA extraction. Human subcutaneous and omental adipose tissue was taken from the abdominal subcutaneous wall, during an operation for vertical banded gastroplasty, from obese female patients. Adipose tissue samples were obtained within 5 min of the tissue being extracted from the patients and frozen immediately in liquid nitrogen. Subjects had fasted overnight prior to surgery. All patients provided informed written consent before inclusion in the study. The study was approved by the Grampian Research Ethics committee.

### CpG island prediction

For each UCP1 ortholog, 5 kb of genomic DNA upstream of the open reading frame start was screened for CGI using a modified version of the CpGLH program (kindly provided by Angie Hinrichs UCSC). Briefly, each sequence is screened for the presence of CG rich regions which fulfill the CGI criteria of at least 200 bp with a minimum of 50% C + G and where the observed number of CpGs divided by the expected number is greater than 0.6 (Gardiner-Garden and Frommer, 1987). The sensitivity of initial screening parameters was modified to identify all possible CGI whilst maintaining the criteria of Gardiner-Garden and Frommer.

### Alignment of homologous promoters

Regions of conservation between cow-human and cow-mouse DNA upstream of UCP1 were determined using rVISTA (Loots et al., 2002) using the AVID alignment algorithm (Bray et al., 2003). For details see Table A2 in Appendix.

### Methylation sensitive restriction digestion

Restriction enzyme digests were performed on 1 μg of genomic DNA extracted from tissues. Primers (Table A3 in Appendix) were designed to cover short and long fragments of the bovine and human CGI in the UCP1 promoter. In the bovine, two restriction enzymes were chosen recognizing the sequence CCGG, *Hpa*II in which digestion is prevented by methylation, and *Msp*I which is not methylation sensitive and acts to correct for incomplete digestion. Two sets of PCR primers were employed, the first with a product size of 288 bp and containing only one CCGG site and a second with product of 407 bp containing five CCGG sites. In the human, two sets of primers amplifying a short (173 bp; one CCGG) and long (426 bp; eight CCGG) region covering part of the human CpG island, were employed. For these digests 1 μg of genomic DNA was incubated with 10 units of *Hpa*II (Fermentas) in the buffer provided (33 mM Tris-acetate, 10 mM Mg-acetate, 66 mM K-acetate, 0.1 mg/ml BSA) in a reaction volume of 50 μl for 4 h at 37°C before the enzyme was heat inactivated at 65°C for 20 min. One microgram aliquots of genomic DNA were also mock-digested under the same conditions but with nuclease free water added instead of *Hpa*II. A final aliquot was digested using 1 unit of *Msp*I (Fermentas) according to the manufacturer’s instructions. The resulting digests were analyzed by quantitative real-time PCR (qRTPCR) using primers for the long and short fragments mentioned above. About 18S mRNA was used as a reference gene with primers (Table A3 in Appendix) which amplify a fragment that does not contain a CCGG motif. The human UCP1 enhancer region does not possess the sequence CCGG so *Tai*I was used which cuts ACGT but is blocked by CpG methylation. Complete digestion was gauged using MnlI which cuts CCTC(N)7.

### Methylated cytosine mapping

Bisulfite conversion of genomic DNA prepared from tissues was carried out essentially as described by Clark et al. (1994). The modified DNA was purified using a desalting column (Promega Wizard DNA Clean-Up system; Promega, Madison, WI, USA) Methylation was quantified by pyrosequencing using Pyro Q-CpG software (Biotage, Charlottesvile, VA, USA) and performed by The Genome Centre, Queen Mary, University of London, Charterhouse Square, London EC1M 6BQ. Primer sequences and descriptions are provided (Table A3 in Appendix), products destined to be pyrosequenced were amplified with 5′-biotin-labeled primers to allow purification before sequencing.

### Real-time PCR

Total RNA was extracted from cultured cells and tissue by use of TRI reagent (Sigma, Poole, UK). Before qRTPCR, samples were treated with RNA-free DNase to remove contaminating genomic or plasmid DNA. Complementary DNA was generated using the cDNA synthesis kit from Qiagen. qRTPCR was performed using Sybr green (Qiagen) according to the manufacturer’s instructions in Rotor Gene 3000 (Corbett Research, Cambridge, UK). The sequences of the primers used for qRTPCR are given in Table A3 in Appendix. Expression levels for all genes were normalized to the internal control 18s rRNA using the ΔΔC_t_ method (Livak and Schmittgen, 2001).

## Results

### Identification of CpG islands

**Figure A1:**
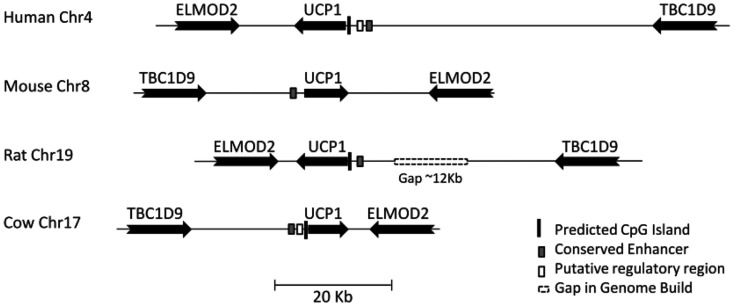
**Gene synteny around the UCP1 locus**. Relative positions of UCP1 orthologs in four representative species showing conservation of synteny. Positions of conserved upstream regions and predicted CpG islands are shown. Gaps in current genome build are shown as hashed boxes. For details see Table A2.

UCP1 homologs from vertebrate species with sufficient genomic data were determined using BLAT at the UCSC genome browser. To ensure that the upstream region of true UCP1 orthologs were compared, the conserved synteny of the UCP1 locus in vertebrates was employed to unequivocally identify the upstream untranslated region of UCP1 in vertebrates. In all species examined the coding region for UCP1 is flanked by TBC1D9 upstream and ELMOD2 downstream (Figure A1 in Appendix). Only those annotated UCP1 genes which were located in the conserved gene triplet TBC1D9-UCP1-ELMOD2 were considered. This resulted in 29 vertebrate UCP1 genes analyzed (see Table A1 in Appendix). A approximately 500 bp sequence with sequence similarity to the human UCP1 enhancer was identified in 20 eutherian mammals but was absent in Marmoset, Pika, Ground Squirrel, Shrew, and Hedgehog (Table 1). The enhancer was also absent from the marsupial Opossum, monotreme Platypus, *Xenopus*, and Zebrafish. A previous study was similarly unable to identify the enhancer in 10 Kb upstream UTR of the marsupial *M. domestica* (Jastroch et al., 2008). The enhancer sequence was within the −5 kb of the UTR except for Tenrec in which the enhancer sequence started at −5.486 Kb (Table A2 in Appendix).

**Table 1:** **Occurrence and position of CpG island, enhancer and putative regulatory region in relation to the start of UCP1 transcription in 27 vertebrate species**.

	CpG	Enhancer (human position −3488) as described in del Mar Gonzalez-Barroso et al. (2000), Jastroch et al. (2008), Shore et al. (2010)	Putative regulatory region (human position −2095)
Human	High stringency	Yes	Yes
Chimp	High stringency	Yes	Yes
Orangutan	High stringency	Yes	Yes
Macaque	High stringency	Yes	Yes
Marmoset	High stringency	X	X
Mouse Lemur	High stringency	Yes	Yes
Tree Shrew	High stringency	Yes	Yes
Pika	Low stringency	X	X
Rabbit	High stringency	Yes	Yes
Guinea pig	High stringency	Yes	Yes
Rat	Low stringency	Yes	X
Mouse	X	Yes	X
Ground Squirrel	High stringency	X	X
Shrew	Low stringency	X	X
Hedgehog	High stringency	X	X
Mega Bat	High stringency	Yes	Yes
Micro Bat	High stringency	Yes	X
Dog	High stringency	Yes	X
Cat	High stringency	Yes	X
Giant Panda	High stringency	Yes	Yes
Horse	High stringency	Yes	Yes
Cow	High stringency	Yes	Yes
Pig	High stringency	yes	X
Tenrec	High stringency	Yes	Yes
Elephant	High stringency	Yes	Yes
Opossom	Low stringency	X	X
Platypus	Low stringency	X	X
*Xenopus*	X	X	X
Zebrafish	Low stringency	X	X

*High stringency regions represent CpG islands identified by the CpGLH algorithm with default settings, low stringency regions represent CpG islands identified by the CpGLH algorithm with relaxed settings (see Materials and Methods). Putative Regulatory Region represents a 500 bp region conserved in some species containing multiple consensus response elements*.

Using a bioinformatic approach, we identified CGI in the UCP1 promoter of different species, fulfilling the criteria originally described by Gardiner-Garden and Frommer (1987). The results clearly demonstrate the existence of a positionally conserved CpG island in the UCP1 5′ UTR in 20 mammalian species (Table 1). By reducing the stringency of the algorithm, an additional five species (rat, shrew, opossum, pika, platypus, and Zebrafish) have identifiable CGI which still fulfill the criteria of Gardiner-Garden and Frommer. From this analysis only two species, Mouse and *Xenopus* do not have a detectable CGI. The positions of the CGI were within 1 kb upstream of the UCP1 translational start site (TSS) except for the European Hedgehog in which the CpG island was located downstream of the TSS.

### CpG methylation of the bovine and human UCP1 CpG island

**Figure 1:**
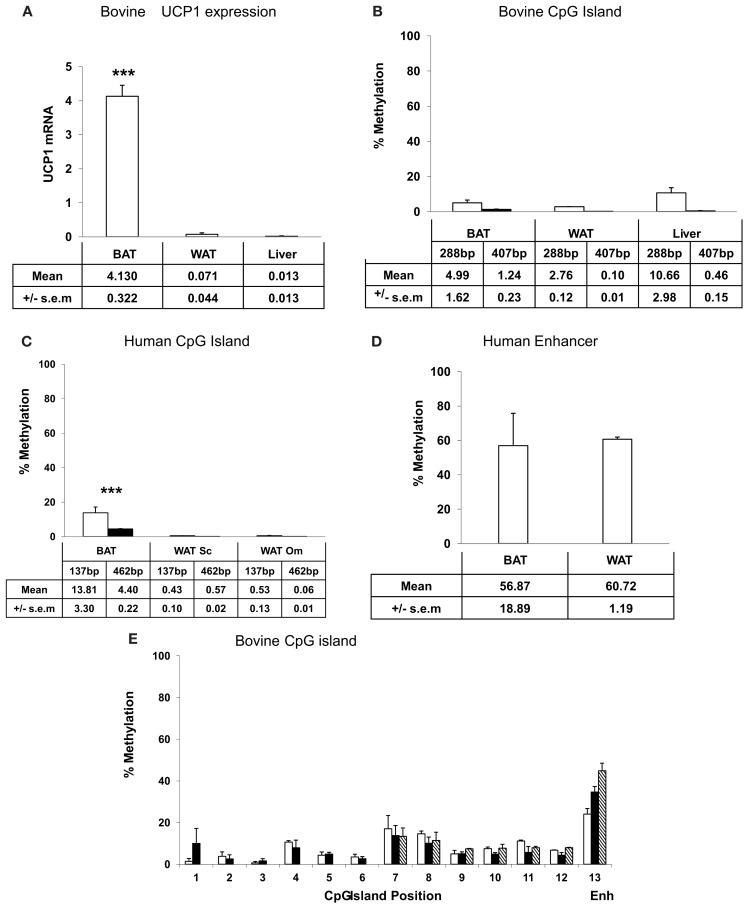
**Expression and UCP1 promoter percentage CpG methylation**. **(A)** bovine UCP1 mRNA expression by qRTPCR. Methylation sensitive restriction digest determination of **(B)** bovine, **(C)** human CpG islands, **(D)** human enhancer, and **(E)** bisulfite mapping determination of the percentage methylation of 12 CpGs within the bovine CpG island, in adipose tissues and liver. UCP1 mRNA **(A)** is expressed relative to ribosomal 18S mRNA. The data are presented as a percentage methylation compared to each respective mock methylated sample for the **(B)** bovine 288 bp (□) and the 407 bp (■) products and **(C)** human 173 bp (□) and the 426 bp (■) products and **(D)** human enhancer (see Materials and Methods). The amount of UCP1 promoter DNA was quantified by qPCR relative to ribosomal 18S DNA. **(E)** CpG dinucleotide methylation in the *Ucp1* proximal promoter in newborn bovine brown (□) and subcutaneous white adipose tissue (■), and liver (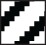). For comparison, values for the mouse enhancer (ENH) BAT, WAT, and liver are presented. DNA was extracted, bisulfite modified, amplified by PCR, and pyrosequenced to determine CpG methylation over positions 1–12 of the *Ucp1* promoter (see Materials and Methods). Missing liver values are due to failed analyses. Values are means ± SEM from at least three replicates except for **(D)** which represents the average of duplicates ± SD *** BAT significantly greater than other tissues (*p* < 0.001).

The high conservation of the CpG island in the proximal UCP1 promoter across evolutionary time in vertebrates suggests that this region may be of regulatory importance. We therefore next examined the methylation state of the proximal promoter in human and the bovine tissues, in order to establish whether BAT-specific expression of UCP1 is dictated by CpG methylation state of the UCP1 promoter. UCP1 mRNA expression in bovine white adipose tissue (WAT, subcutaneous), BAT (perirenal), and liver were determined by qRTPCR. BAT had significantly greater (200-fold) UCP1 expression than WAT or liver (Figure 1A; *p* < 0.001). The high expression of UCP1 in BAT was not unexpected since these samples were taken shortly after birth (8 h) and previous studies, including our own in ruminants, have shown that UCP1 expression is at its highest around parturition in response to the cold extrauterine environment (Symonds and Lomax, 1992). Previous studies have demonstrated that UCP1 expression is high in human fetal BAT (Gavrilova et al., 1988).

Methylation sensitive restriction digests were carried out on genomic DNA extracted from neonatal bovine BAT, subcutaneous WAT, and liver, fetal human BAT, and adult human WAT, (omental and subcutaneous) to determine differences in methylation state between the tissues. Methylation of the bovine proximal promoter CpG island was low in all tissues with a 407 bp product being less than 2% methylated and a 288 bp product less than 12%. (Figure 1B). There was no significant difference in methylation state of the CpG island between bovine tissues. It was expected that the 407 bp fragment would be more susceptible to methylation sensitive digestion as this contained more restriction sites, increasing the probability that a methylated site would be encountered by the enzyme. In the human proximal promoter CpG island, methylation state of fetal BAT was also low (<14%) but was significantly (*p* < 0.05) higher (173 bp product, 14%: 426 bp product, 4% methylated) than WAT from both depots which were un-methylated (Figure 1C). A similar methylation sensitive restriction digestion approach (see Materials and Methods) demonstrated that the methylation state of a region of the human enhancer was much higher (55–60%) than the proximal promoter CpG island (Figure 1D). The primers amplified a region that contains this sequence which also lies at the consensus CRE homologous to CRE3 in the mouse.

We next employed bisulfite mapping in order to confirm the apparent low levels of methylation in the bovine CpG island, in the bovine tissues. CGI are difficult to analyze using PCR bisulfite mapping due to the problem of designing primers and although we attempted to amplify 44 CpGs in and around the bovine CpG island we were only able to produce reliable results for 12 CpGs. In agreement with the methylation sensitive restriction digests, all of these CpGs had methylation levels less than 20% with the majority below 10% with no significant differences between the tissue types (Figure 1E). For comparison, the values for CpG methylation of the mouse enhancer around CRE3 determined by bisulfite mapping in our previous studies (Shore et al., 2010) have been included in Figure 1E to emphasize the relatively low methylation state of the bovine CpG island. There was insufficient human BAT to carry out a similar bisulfite mapping analysis.

### The position of a conserved 5′ upstream enhancer region and a putative regulatory region in the promoter of UCP1 in vertebrates

Since methylation CpG state of the UCP1 promoter was unable to explain brown adipose-specific expression, we next turned our attention to the bioinformatics analysis of the promoter region. Conservation of a 320 bp enhancer in a 10 Kb region upstream of the UCP1 TSS has been previously reported in eutherians, including the Afrotherian species but not in the marsupial *M. domestica*, (Jastroch et al., 2008). We extended this study to include non-mammalian vertebrates (Table 1). Surprisingly, although we could detect the enhancer box in the 10 kb sequence upstream of the TSS in 20 eutherian species, it was not present in five eutherians (Marmoset, Pika, Ground Squirrel, Shrew, Hedgehog) despite BAT-specific UCP1 expression in these species. The low coverage (approximately 2×) of four of these (Pika, Ground Squirrel, Shrew and Hedgehog) is likely to be insufficient to confidently conclude the lack of this enhancer. However Marmoset has increased coverage (6×) and provides greater confidence of the loss of enhancer in mammalian species. Within the marmoset genome the nearest gap upstream of the UCP1 gene is estimated to be 54,083 bp upstream, suggesting that the lack of predicted enhancer is not due to missing sequence data. As expected the enhancer box was not detected in the marsupial Opossum, the monotreme, Platypus, or non-mammalian vertebrates (*Xenopus*, Zebrafish). Within the mammalian species possessing a 5′ distal enhancer there was remarkable conservation of response element sequences that have been shown to regulate UCP1 transcription in rodent studies, as previously noted by Jastroch et al. (2008; Figures A3–A5 in Appendix). The enhancer sequence was within the −5 kb of the UTR except for Tenrec in which the enhancer sequence started at −5.486 Kb (Table A2 in Appendix). The presence of a conserved enhancer sequence upstream of pig UCP1 is possibly unexpected. The UCP1 gene was predicted to have become a pseudogene approximately 20 million years ago (Berg et al., 2006). If the sole role of the enhancer is associated with UCP1 expression, it would be predicted that following pseudogenization that purifying selection of UCP1 enhancer would be relaxed, resulting in degeneration of conservation by accumulation of mutations. However, the pig enhancer remains well conserved. Pairwise percent identify of Human-cow enhancer is 78.5% and is only slightly lower in Human-pig (75.9%). This suggests a possible additional role for the enhancer in pig or that the expression of a truncated form of UCP1 is transcribed in pig.

A second conserved putative regulatory region of approximately 500 bp was noted (Human −2095; usually placed 2200–2700 bp upstream of the TSS in most species) which although present in 14 of the eutherian species, was absent in the nine vertebrate species that we could not find the enhancer, with the exception of rodents (Table 1; Table A2 in Appendix; Figures A3 and A4 in Appendix).

**Figure 2:**
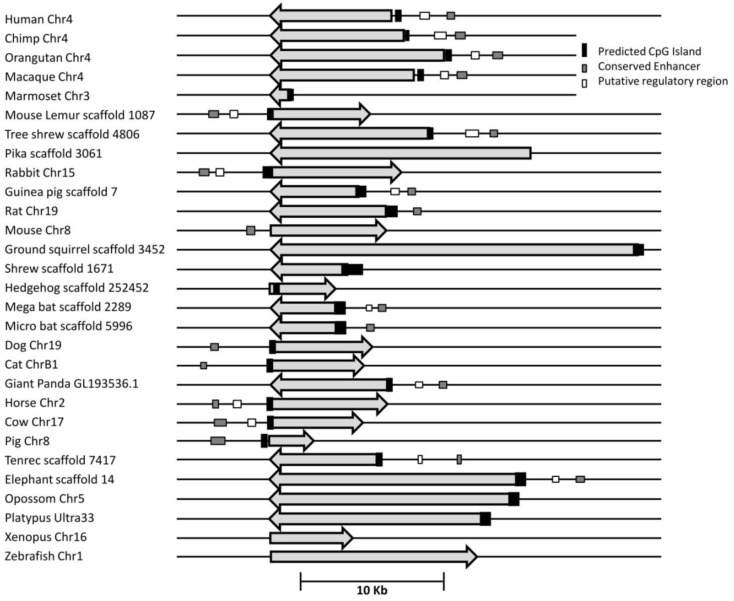
**Map of the relative positions of the conserved enhancer, putative regulatory region and predicted CpG island in the UCP1 promoter of 29 species**. All genes are shown in 5′–3′ orientation. Arrow represent the region of the UCP1 coding sequence. Differences in arrow length are likely to reflect relative differences in intron sizes.

Pairwise comparison of bovine-mouse, or bovine-human promoters using Rvista (Loots et al., 2002) highlighted this conserved putative regulatory region between the human and bovine approximately 2.5 Kb upstream, but not between bovine and mouse (Figure 2). As expected, a highly conserved peak is visible at approximately −3.6 Kb within the conserved enhancer region and contained the conserved transcription factor binding sites previously mentioned above. A second conserved region approximately −1.1 to −1.6 kb is conserved between bovine and human but is missing in mouse and rat genomes. The putative regulatory region also contained a number of conserved transcription factor binding sites (CEBP, CREB, DR1, DR3, DR4, PPAR) suggesting the presence of control elements that may be important in regulating species-specific UCP1 expression.

## Discussion

The recent discovery of BAT in adult humans has excited interest in combating obesity by stimulating the expression and activity of UCP1 in brown adipocytes in order to increase energy expenditure. In order to manipulate energy expenditure it is necessary to understand the precise transcriptional regulation of UCP1 and although there have been recent advances in the transcriptional factors and co-regulators required for activating the brown adipogenic gene expression, the mechanisms responsible for the species-specific and tissue-specific expression of UCP1 are unknown. The vast majority of studies have been carried out in rodents which retain neonatal brown depots into adulthood. In humans neonates, significant amounts of BAT are found in the perirenal and axillary depots, disappearing in adults but being replaced by the recently discovered supraclavicular depots. We and others have reported a similar developmental disappearance of BAT from the perirenal depot in ruminants (Lomax et al., 2007). We have proposed that tissue-specific expression may be dictated by the methylation of specific CpGs in cyclic AMP response elements in the UCP1 enhancer unit (Shore et al., 2010). An alternative suggestion is that methylation of CpGs in CGI in the promoter may confer tissue-specific expression of UCP1 (Kiskinis et al., 2007).

Using a bioinformatic approach we were able to identify a CpG island conserved across 26 of 28 mammalian including marsupials and monotremes (Figure 2). Additionally a CpG island can be identified upstream of the Zebrafish UCP1 transcription start site suggesting a more ancient origin and that this CpG island predates the divergence of mammals. In the context of the evolution of the CGI in the UCP1 promoter, it is therefore unlikely that the retention of the CpG island is related to the acquisition of BAT-specific expression since this is a feature only of mammals. This conclusion was supported by our study using methyl sensitive restriction digestion and qPCR which demonstrates that the methylation state of the bovine CpG island does not appear to account for the differential expression of UCP1 shown by qPCR between BAT and WAT and that the CpG island remains essentially demethylated in BAT, WAT, and liver tissues regardless of the level UCP1 expression. These low methylation states were confirmed by pyrosequencing analysis of the region. Though it is possible that some of the unsequenced CpGs show differential methylation levels, we show that there is not a wide ranging difference in methylation state compared with differences in UCP1 expression. These findings were confirmed in the human tissues where there were also low levels of methylation and no apparent difference between fetal BAT and adult WAT promoter methylation despite well documented difference in UCP1 expression between these tissues (Lean and James, 1986).

We have previously observed in mice that CpG dinucleotide methylation of the *Ucp1* distal enhancer exhibits tissue-specific patterns in murine tissue and cell lines and suggested that adipose tissue-specific *Ucp1* expression involves demethylation of CpG dinucleotides found in regulatory CREs in the *Ucp1* enhancer, as well as modification of histone tails (Shore et al., 2010). The control of UCP1 expression by a complex series of response elements in the 5′ distal enhancer has been studied in the rodent and human promoter (del Mar Gonzalez-Barroso et al., 2000; Rim and Kozak, 2002) where this enhancer is necessary for both response to drugs and tissue-specific expression. However the observation that marsupial *M. domestica* expresses UCP1 in response to beta adrenergic stimulation despite there being no identifiable enhancer suggests that other regulatory mechanisms exist (Jastroch et al., 2008). We confirmed this observation and have demonstrated that the enhancer is also absent from the other species Marmoset, Pika, Ground Squirrel, Shrew, and Hedgehog despite evidence that of BAT-specific expression of UCP1 in these species (Rothwell and Stock, 1985; Loncar, 1990; Liu et al., 1998; Suzuki et al., 2006; Kitao et al., 2007). All of the nine species lacking an identifiable enhancer also lacked the putative regulatory region but further studies are necessary to characterize this region (Figure 2). Taken together the results do not support a role for either CpG island methylation or the presence of an enhancer unit, in tissue-specific regulation of UCP1 expression.

**Figure A2:**
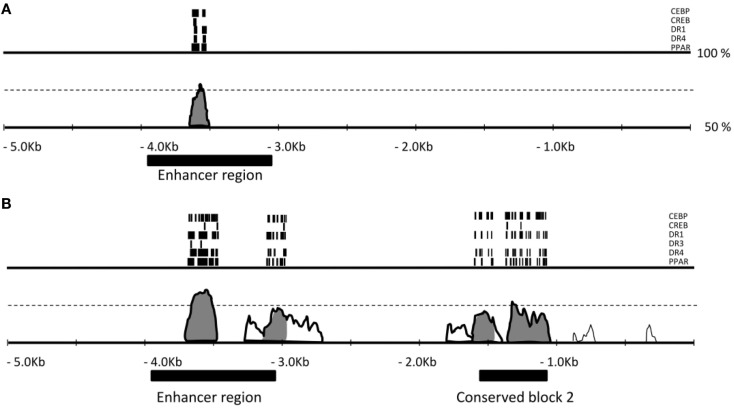
**Evolutionary conservation of the regions of the UCP1 5′ UTR enhancer and putative regulatory region**. Pairwise comparison of cow-mouse **(A)** and cow-human **(B)** genomic DNA 5 Kb upstream of UCP1 gene rVISTA (Loots et al., 2002) using the AVID alignment algorithm (Bray et al., 2003). Conserved regions (>70% conserved in 100 bp window are shaded). A highly conserved peak is visible at approximately −3.6 Kb within the conserved enhancer region. A second conserved region approximately −1.1 to −1.6 kb is conserved between cattle and human but is missing in the mouse genome. Within peaks of sequence similarity are a number of conserved transcription factor binding sites of interest (CEBP, CREB, DR1, DR3, DR4, PPAR) marked with the bars.

**Figure A3:**
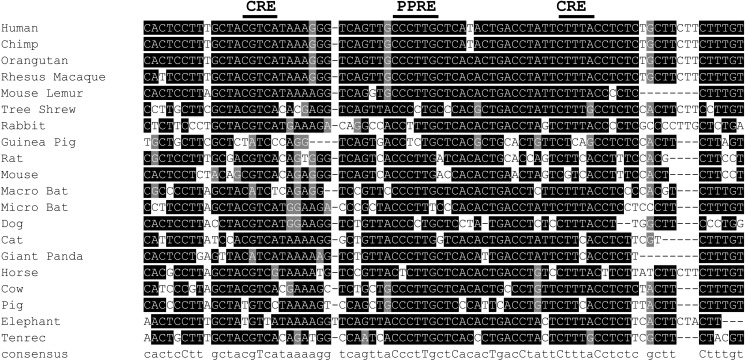
**Partial alignment of conserved enhancer region in 20 vertebrate species, approximately −3800 bp of human UCP1**. For genome coordinates and full alignment, see Appendix.

**Figure A4:**
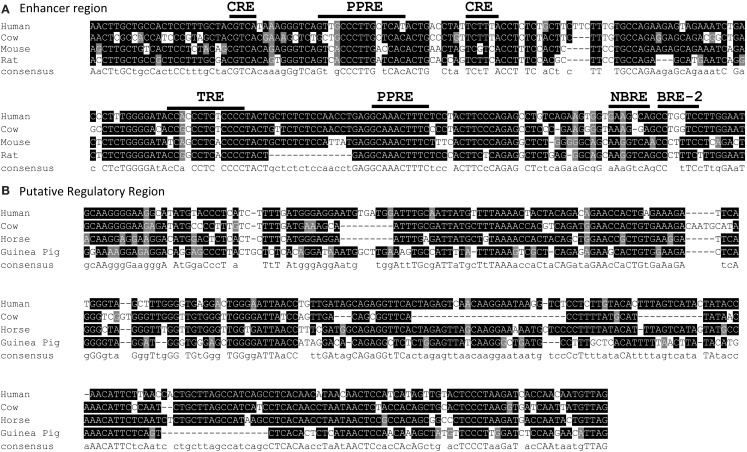
**(A)** Alignment of conserved enhancer region in human, bovine, rat, and mouse, approximately −3800 bp of human UCP1. Positions of known transcription factor binding sites taken from Jastroch et al. (2008) **(B)** Partial alignment of conserved putative regulatory region (PRR) approximately −2200 to −2700 bp of human UCP1. For genome coordinates and full alignment, see Appendix.

**Figure A5:**
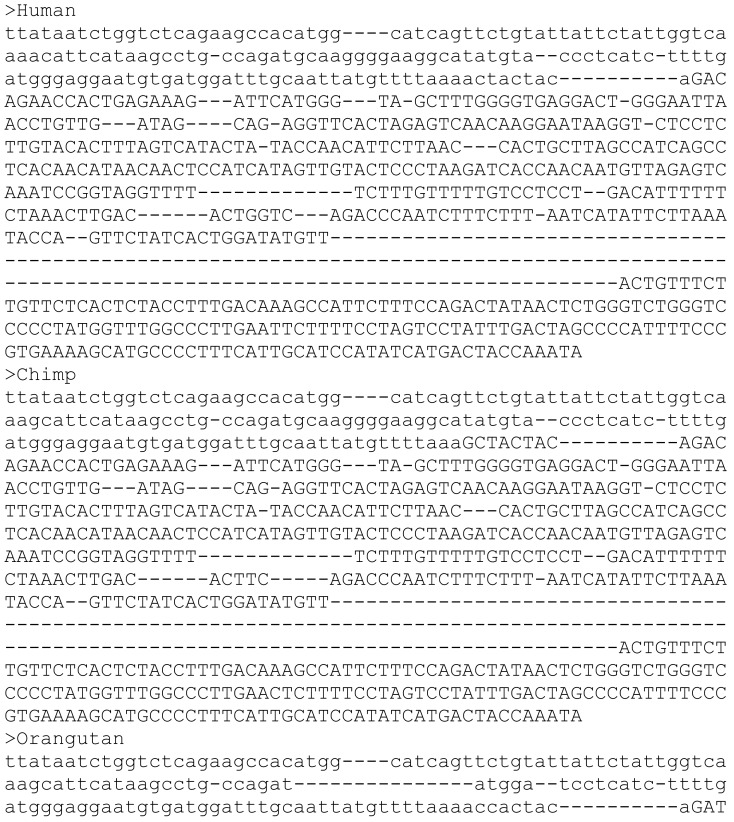

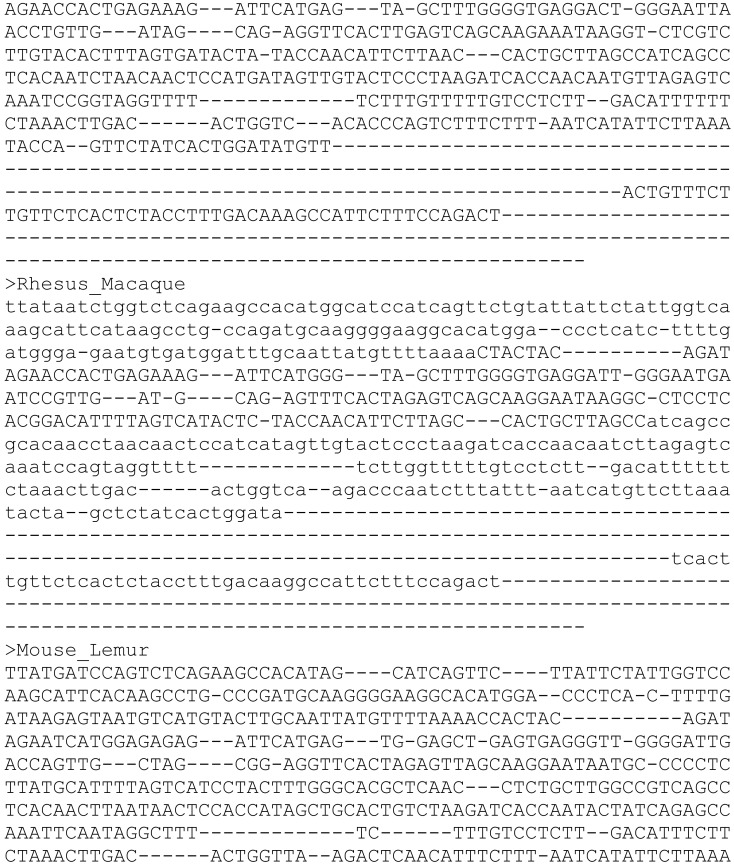

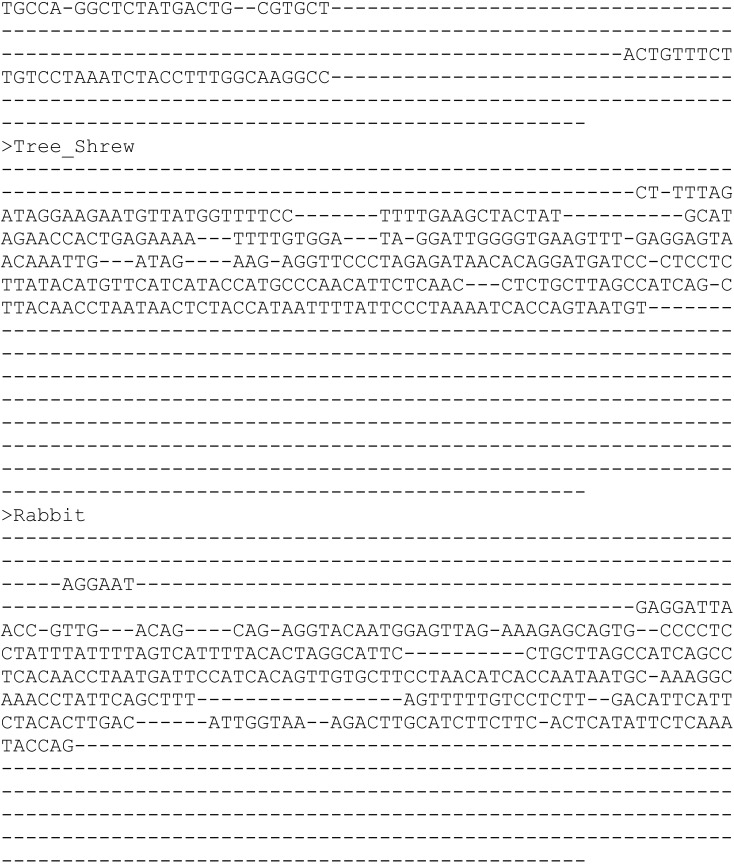

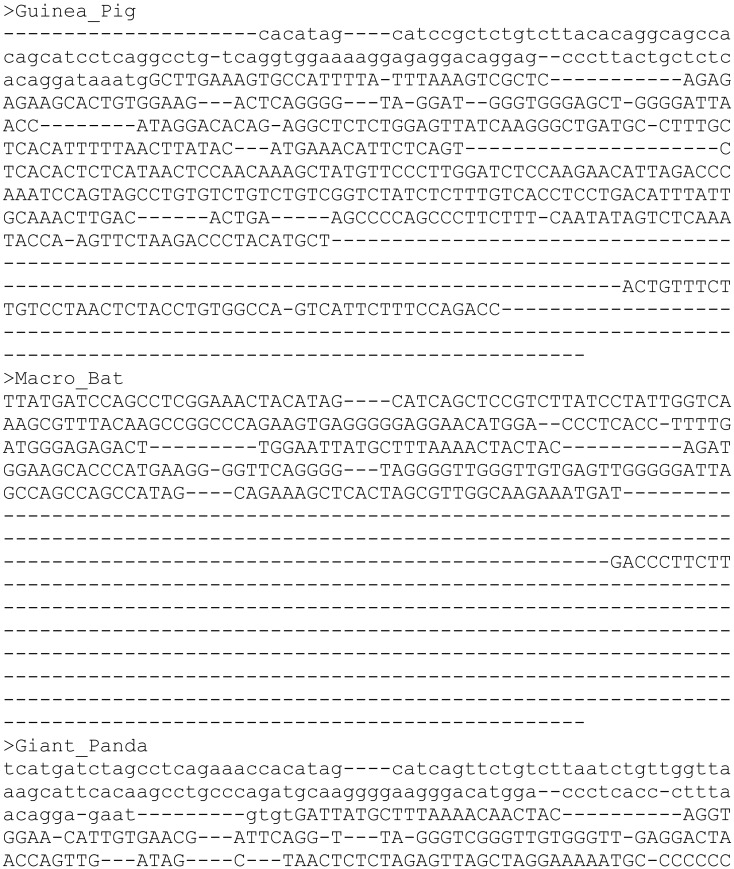

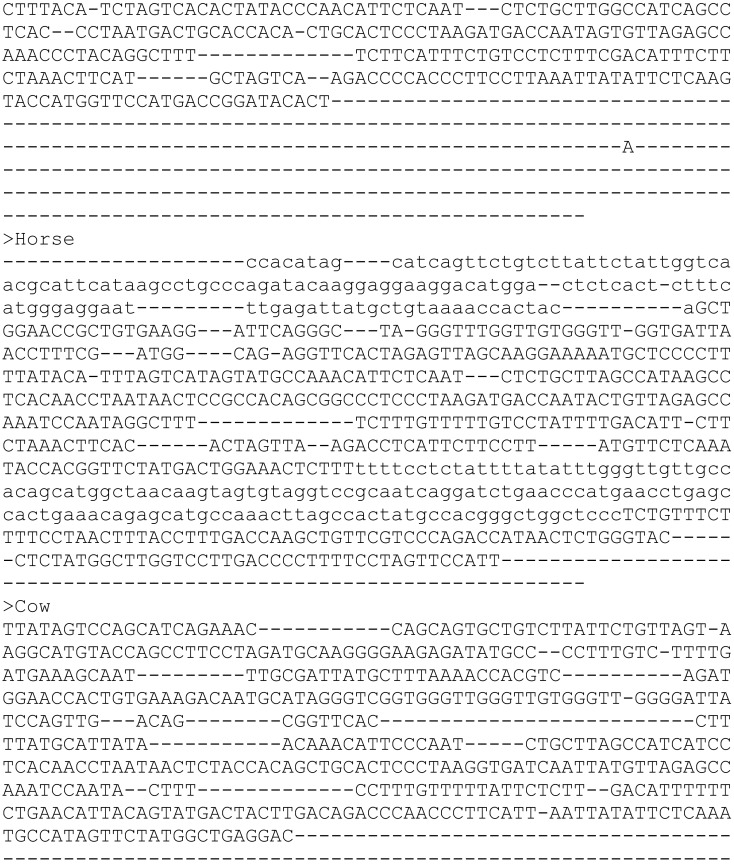

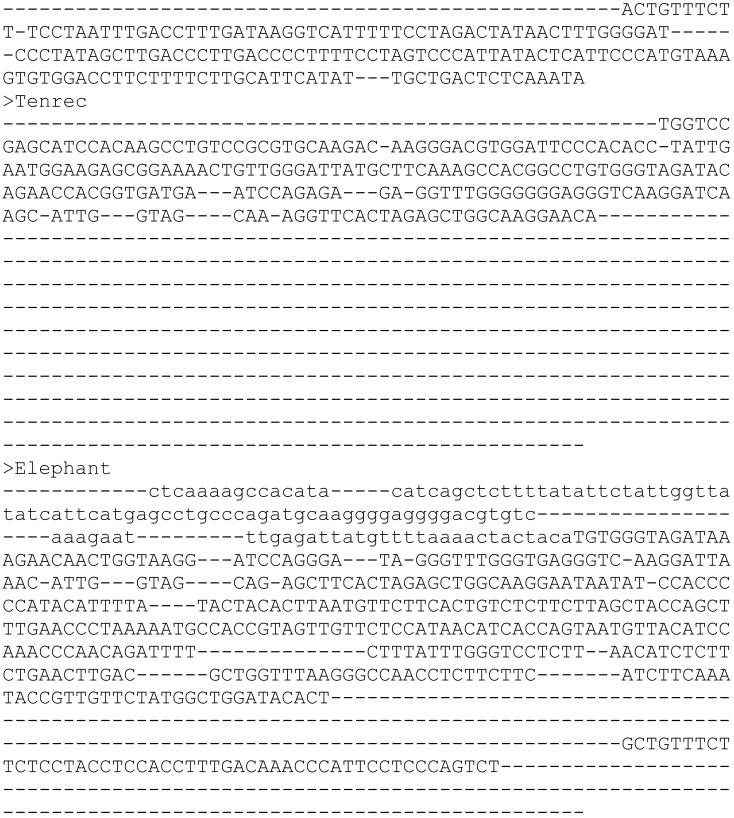
**Sequence of the conserved enhancer region in 20 vertebrate species**.

Our previous study suggested that the loss of adrenergic stimulation of UCP1 expression in perirenal adipose tissue from newborn ruminants is associated with a decrease in the expression of the PPARγ coactivator PGC1α (Lomax et al., 2007) suggesting that the transcriptional machinery in ruminants may fail to activate the enhancer after birth. In rodents cAMP response elements are present in both the enhancer and the proximal promoter (Rim and Kozak, 2002). We have previously demonstrated using mouse cell lines, that the exact combination of transcription factors binding to cAMP response elements, governs the brown adipocyte-specific expression of PGC1α and UCP1, in response to cAMP stimulation (Karamanlidis et al., 2007; Karamitri et al., 2009). Further studies in rodents have also suggested synergistic relationships between the transcriptional factors, PPARγ, PPARα, and PGC1α in brown adipogenesis (Rim et al., 2004; Xue et al., 2005). Therefore, the species differences in the presence of an enhancer and the patterns of brown fat thermogenesis may depend on the specific combinations and trans-activational prowess of transcription factors, rather than the exact structure of 5′ upstream elements. Further studies are required to identify the role of transcription factors activating the CREB and PPAR response elements identified in the bovine PRR (Figure 2; Figure A2 in Appendix) in the regulation of thermogenesis in different species.

## Conclusion

The results presented here demonstrate that mammals possess a highly conserved CpG island close to the transcription start site on the UCP1 promoter but that methylation of the CpG island does not appear to account for tissue-specific expression of UCP1 in these species. The evolution of the enhancer element appears to be separate from the thermoregulatory function of BAT with species lacking an enhancer being able to increase UCP1 expression in response to cold stimulus, or as in the pig, retain the enhancer despite UCP1 becoming a pseudogene. Therefore, although previous studies in rodents have proposed that regulation of UCP1 expression is mainly targeted at response elements in a complex enhancer, a comparative approach suggests that despite clear evidence of conservation of regulatory elements in the UCP1 5′ untranslated region, this does not appear to be related to species- or tissues-specific expression of UCP1. This suggests that the control of mammalian thermogenesis in BAT is not simply due to the evolution of UCP1 promoter elements but the result of a complex interplay between transcriptional regulators and response elements on the UCP1 promoter.

## Conflict of Interest Statement

The authors declare that the research was conducted in the absence of any commercial or financial relationships that could be construed as a potential conflict of interest.

## Appendix

**Table A1:** **Genome builds of species investigate**.

Common name	Latin name	Genome build
Human	Homo sapiens	March 2006 hg18
Chimp	Pan troglodytes	March 2006 panTro2
Orangutan	*Pongo pygmaeus* abelii	July 2007 ponAbe2
Rhesus	*Macaca mulatta*	January 2006 rheMac2
Marmoset	*Callithrix jacchus*	June 2007 calJac1
Mouse lemur	*Microcebus murinus*	June 2003 micMur1
TreeShrew	*Tupaia belangeri*	December 2006 tupBel1
Pika	*Ochotona princeps*	July 2008 ochPri2
Rabbit	*Oryctolagus cuniculus*	May 2005 oryCun1
Guinea pig	*Cavia porcellus*	February 2008 cavPor3
Rat	*Rattus norvegicus*	November 2004 rn4
Mouse	*Mus musculus*	July 2007 mm9
Ground squirrel	*Spermophilus tridecemlineatus*	February 2008 speTri1
Shrew	*Sorex araneus*	June 2006 sorAra1
Hedgehog	*Erinaceus europaeus*	June 2006 eriEur1
Megabat	*Pteropus vampyrus*	July 2008 pteVam1
Microbat	*Myotis lucifugus*	March 2006 myoLuc1
Dog	*Canis lupus* familiaris	May 2005 canFam2
Cat	*Felis catus*	March 2006 felCat3
Giant panda	*Ailuropoda melanoleuca*	AilMel 1.0 December 2009
horse	*Equus caballus*	September 2007 equCab2
Cow	*Bos taurus*	November 2009 bosTau6
Pig	*Sus scrofa*	SGSC Sscrofa9.2
Tenrec	*Echinops telfairi*	July 2005 echTel1
Elephant	*Loxodonta africana*	July 2008 loxAfr2
Opossum	*Monodelphis domestica*	January 2006 monDom4
Platypus	*Ornithorhynchus anatinus*	March 2007 ornAna1
Xenopus tropicalis	*Xenopus tropicalis*	August 2005 xenTro2
Zebrafish	*Danio rerio*	July 2007 danRer5

**Table A2:** **Gene coordinates and start/stop positions relative to the UCP1 transcriptional start site of enhancer region and putative regulatory region (PRR) from the 29 vertebrate species examined**.

Species	Genome build	Chromosome	CpG identified	UCP1 ortholog gene or prediction name	Gene start	Gene end	Gene strand	Enhancer start coordinate	Enhancer stop coordinate	PRR start coordinate	PRR stop coordinate	Enhancer start relative position	Enhancer stop relative position	PRR start relative position	PRR stop relative position
Human	Hg19	4	High stringency	NM_021833	141481052	141489959	−	141493442	141493950	141492054	141492731	−3483	−3991	−2095	−2772
Chimp	PanTro2	4	High stringency	N-Scanchr4.145.006.a	144322263	144332101	−	144335822	144336333	144334440	144335115	−3721	−4232	−2339	−3014
Orangutan	ponAbe2	4	High stringency	N-Scanchr4.983.1	145986899	145999241	−	146002758	146003264	146001158	146001693	−3517	−4023	−1917	−2452
Macaque	rheMac2	5	High stringency	N-Scanchr5.134.002.a	133012794	133023077	−	133026214	133026725	133024970	133025515	−3137	−3648	−1893	−2438
Marmoset	calJac3	3	High stringency	N-Scanchr3.6.016.a	52372048	52373243	+	n/d	n/d	n/d	n/d	n/d	n/d	n/d	n/d
Mouse Lemur	micMur1	GeneScaffold_1087	High stringency	ENSMICG00000008999	4785	11471	+	15010	15507	13790	14315	−3539	−4036	−2319	−2844
Tree Shrew	tupBel1	GeneScaffold_4806	High stringency	ENSTBEG00000000042	25827	37487	−	41768	42362	40139	40400	−4281	−4875	−2652	−2913
Pika	ochPri1	GeneScaffold_3061	Low stringency	ENSOPRG00000004634	482498	500869	−	n/d	n/d	n/d	n/d	n/d	n/d	n/d	n/d
Rabbit	oryCun1	15	High stringency	ENSOCUG00000002297	24001193	24010449	+	23996292	23996791	23997600	23997868	−4901	−4402	−3593	−3325
Guinea pig	cavPor3	scaffold_7	High stringency	ENSCPOG00000001969	19528283	19534394	−	19537941	19538470	19536829	19537347	−3547	−4076	−2435	−2953
Rat	rn4	19	Low stringency	NM 012682	26527548	26535621	−	26537717	26538206	n/d	n/d	−2096	−2585	n/d	n/d
Mouse	mm9	8	X	NM_009463.3	85814247	85822355	+	85811607	85812107	n/d	n/d	−2640	−2140	n/d	n/d
Ground Squirrel	speTri1	GeneScaffold_3452	High stringency	ENSSTOG00000003104	313456	339106	−	n/d	n/d	n/d	n/d	n/d	n/d	n/d	n/d
Shrew	sorAri	GeneScaffold_1671	Low stringency	ENSSARG00000000985	5257	10644	−	n/d	n/d	n/d	n/d	n/d	n/d	n/d	n/d
Hedgehog	eriEur1	scaffold_252 452	High stringency	ENSEEUG00000005182	241	4846	+	n/d	n/d	n/d	n/d	n/d	n/d	n/d	n/d
Mega Bat	pteVam1	GeneScaffold_2289	High stringency	ENSPVAG00000016781	114991	119682	−	122609	123123	121691	121957	−2927	−3441	−2009	−2275
Micro Bat	myoLuc1	GeneScaffold_5996	High stringency	ENSMLUG00000009574	63872	68908	−	70701	71160	n/d	n/d	−1793	−2252	n/d	n/d
Dog	canFam2	19	High stringency	NM 001003046	5283508	5290786	+	5279305	5279811	n/d	n/d	−4203	−3697	n/d	n/d
Cat	felCat4	B1	High stringency	N-ScanchrB1.11.033.a	104387901	104394541	+	104383258	104383501	n/d	n/d	−4643	−4400	n/d	n/d
Giant panda	ailMel1	GL193536.1	High stringency	ENSAMEG00000002869	40351	48686	−	52280	52804	50675	51166	−3594	−4118	−1989	−2480
Horse	equCab2	2	High stringency	ENSECAG00000026962	90911780	90919989	+	90907788	90908113	90908976	90909697	−3992	−3667	−2804	−2083
Cow	bosTau6	17	High stringency	NM_001166528	17467450	17473822	+	17463388	17464315	17465820	17466444	−4062	−3135	−1630	−1006
Pig	susScr2	8	High stringency	N-Scanchr8.8.037.a	74588882	74591948	+	74586513	74587108	n/d	n/d	−2369	−4840	n/d	n/d
Tenrec	echTel1	GeneScaffold_7417	High stringency	ENSETEG00000010924	63037	70485	−	75971	76373	73472	73688	−5486	−5888	−2987	−3203
Elephant	loxAfr3	scaffold 14	High stringency	ENSLAFG00000007077	56118058	56135088	−	56139273	56139912	56137582	56138101	−4185	−4824	−2494	−3013
Opossom	monDom 5	5	Low stringency	ENSMODG00000000172	138908757	138925466	+	n/d	n/d	n/d	n/d	n/d	n/d	n/d	n/d
Platypus	ornAna1	Ultra33	Low stringency	ENSOANG00000015294	877070	891895	−	n/d	n/d	n/d	n/d	n/d	n/d	n/d	n/d
*Xenopus*	xenTro2	16	X	NM 001113882.1	1007554	1013326	+	n/d	n/d	n/d	n/d	n/d	n/d	n/d	n/d
Zebrafish	danRer7	1	Low stringency	NM 199523.2	53870179	53884602	+	n/d	n/d	n/d	n/d	n/d	n/d	n/d	n/d

*n/d indicates that a conserved region could not be identified*.

**Table A3:** **Primer sequences for QPCR quantification of mRNA and methylation sensitive restriction digests, bisulfite specific PCR, and pyrosequencing**.

Primer name	Primer sequence (5′–3′)	PCR annealing temp (°C)	*CpG* positions
**QPCR QUANTIFICATION OF mRNA**			
Bov UCP1F	CACTAGGGAAGGACCGTCAG	55	
Bov UCP1 R	TTCCCGAGGAGGACTAGGTT	55	
Hom UCP1 F	TGCCCAACTGTGCAATGAA	56	
Hom UCP1 R	TCGCAAGAAGGAAGGTACCAA		
18S F	GTAACCCGTTGAACCCCATT	56	
18S R	CCATCCAATCGGTAGTAGCG	56	
**QPCR QUANTIFICATION OF METHYLATION SENSITIVE RESTRICTION DIGESTS**			
Bov Long F	GCATCGAGGGTAGAGCGTAG	56	
Bov Long R	GTGTCCCACCATCCTGACTC	56	
Bov Short F	TCCGGCGATATAAGTCATCC	56	
Bov Short R	CTCTCCGACTTCTGCCCAGT	56	
Hom L and S F	CCAAAGGGTGACAGAAGGTG	56	
Hom Long R	CAGCAAACCCGATTTCTGTT	56	
Hom Short R	GTCCCTCCCATTCCCATTC	56	
**BISULFITE SPECIFIC PCR (PRIOR TO PYROSEQUENCING)**			
Bov Pyro F	GGAGGTAGGTAGGGGGTTGT	56	1,2,3,4,5,6
Bov Pyro R	BIO-AAAACCTACCCCCCAAAACAC	56	1,2,3,4,5,6
Bov Pyro F	GGGGATTAGGGTTTTAGTTTTAAAGGT	52	7,8,9,10
Bov Pyro R	BIO-CCCCCACCTACCACCTAAA	52	7,8,9,10
Bov Pyro F	GTGGTGTTTAGTGGGAAGGTGATTATG	52	11 and 12
Bov Pyro R	BIO-ACCTTTAAAACTAAAACCCTAATCCC	52	11 and 12
Mouse Pyro F	GATGTTTTTGTGGTTTGAGTGTA	58	1,2,3,4
Mouse Pyro R	BIO-TCCCCAAAAAATCTAATTTCTAC	58	1,2,3,4
Mouse Pyro F	TTTTGGGGGTAGTAAGGTTAAT	53.3	5 and 6
Mouse Pyro R	BIO-TATTACCCAACAAAAACTTTCC	53.3	5 and 6
**PYROSEQUENCING PRIMERS**			
Bov Pyro S1	TTTAGAGTTAGGGTTGGTTA		1,2,3,4,5,6
Bov Pyro S2	TGTTTTGTTTGGTTTTTTAT		7,8,9,10
Bov Pyro S3	GGTTGTTATTTTAGTTGAGA		11 and 12
Mouse Pyro S1	TTGTGAAATGAGTGAGTAA		1
Mouse Pyro S2	TGGTGTTTTATATTTTAAG		2
Mouse Pyro S3	TAGGTAAGTGAAGTTTGTTG		3
Mouse Pyro S4	ATTTTTGATTATATTGAATT		4
Mouse Pyro 5–6	TTTTTTGTTTTGAGTTGATA		5 and 6

*BIO indicates biotinylation and CpG position represents CpG dinucleotides successfully pyrosequenced in the bovine (Bov) and human (Hom) proximal promoters*.
